# Beyond hemostasis: a snake venom serine protease with potassium channel blocking and potential antitumor activities

**DOI:** 10.1038/s41598-020-61258-x

**Published:** 2020-03-11

**Authors:** Johara Boldrini-França, Ernesto Lopes Pinheiro-Junior, Steve Peigneur, Manuela Berto Pucca, Felipe Augusto Cerni, Rafael Junqueira Borges, Tássia Rafaella Costa, Sante Emmanuel Imai Carone, Marcos Roberto de Mattos Fontes, Suely Vilela Sampaio, Eliane Candiani Arantes, Jan Tytgat

**Affiliations:** 1 School of Pharmaceutical Sciences of Ribeirão Preto, University of São Paulo, Av. do Café s/n°, 14040-903 Ribeirão Preto, SP Brazil; 2 Toxicology and Pharmacology, KU Leuven, O&N II Herestraat 49, PO 922, 3000 Leuven, Belgium; 3 Medical School of Roraima, Federal University of Roraima, Av. Capitão Ene Garcez, 2413, Bairro Aeroporto, 69310-970 Boa Vista, RR Brazil; 4 Institute of Biosciences, São Paulo State University (UNESP), Rua Prof. Dr. Antonio Celso Wagner Zanin, 250, 18618-689 Botucatu, SP Brazil; 5 University of Vila Velha, Av. Comissário José Dantas de Melo, 21, Boa Vista II, 29102-920 Vila Velha, ES Brazil

**Keywords:** Enzymes, Potassium channels

## Abstract

Snake venom serine proteases (SVSPs) are complex and multifunctional enzymes, acting primarily on hemostasis. In this work, we report the hitherto unknown inhibitory effect of a SVSP, named collinein-1, isolated from the venom of *Crotalus durissus collilineatus*, on a cancer-relevant voltage-gated potassium channel (hEAG1). Among 12 voltage-gated ion channels tested, collinein-1 selectively inhibited hEAG1 currents, with a mechanism independent of its enzymatic activity. Corroboratively, we demonstrated that collinein-1 reduced the viability of human breast cancer cell line MCF7 (high expression of hEAG1), but does not affect the liver carcinoma and the non-tumorigenic epithelial breast cell lines (HepG2 and MCF10A, respectively), which present low expression of hEAG1. In order to obtain both functional and structural validation of this unexpected discovery, where an unusually large ligand acts as an inhibitor of an ion channel, a recombinant and catalytically inactive mutant of collinein-1 (His43Arg) was produced and found to preserve its capability to inhibit hEAG1. A molecular docking model was proposed in which Arg79 of the SVSP 99-loop interacts directly with the potassium selectivity filter of the hEAG1 channel.

## Introduction

Voltage-gated potassium channels (Kv) are involved in a diversity of physiological processes, such as smooth muscle contraction^1^, cell volume control^2^, cell cycle progression^3^, cardiac repolarization^4^, and proliferation of tumor cells^5^. Several animal venom-related toxins are known to modulate Kv channel activity either by blocking^6^ the ion selective pore or by modulating^7^ the channel gating (*i.e*. opening and closing mechanisms). Pore blocking toxins bind to the external opening of the pore or to the internal cavity underneath the channel selectivity filter^8^, while gating modifiers induce conformational changes in the voltage-sensing domain of the channel, affecting the kinetics of channel opening and closing^9,10^. Most of the Kv-ligand toxins known to date are found in scorpion, spider, cone snail and sea anemone venoms^10–13^; only relatively few snake venom toxins are known to interfere with ion channel activity, such as dendrotoxins, the B chain of β-bungarotoxin, and crotamine, acting on Kv channels^14–18^.

Snake venom serine proteases (SVSPs) comprise a group of extensively studied toxins, widely found in the venom of terrestrial snakes from Viperidae, Elapidae, and Crotalidae families. Snake venom thrombin-like enzymes (SVTLEs) are the prevalent class of serine proteases from Viperidae venoms and present similar activity to that of human thrombin^19–21^. SVSPs may be considered multifunctional toxins due to their broad substrate specificity and can thus act on different systems of the prey or victim organisms^22^. Therefore, the investigation of the intrinsic pathways involved in the variety of biological activities of these molecules may contribute to expanding their potential applications. To the best of our knowledge, the effect of SVSPs on ion channel activity has never been described before in the scientific literature.

This study reports for the first time the ion channel blocking activity of a SVTLE from *Crotalus durissus collilineatus* venom, named collinein-1^23^, on the oncogenic *ether-a-go-go 1* voltage-gated potassium channel (hEAG1, Kv10.1, *KCNH1*), revealing a completely novel target for SVSPs. This unexpected property is important for a better understanding and unravelling of the complex and pluripotent pharmacology of SVTLEs, and may open perspectives in terms of applicability, particularly in the field of oncology.

## Collinein-1 blocks hEAG1 channels by a catalytic triad-independent mechanism

**Figure 1:**
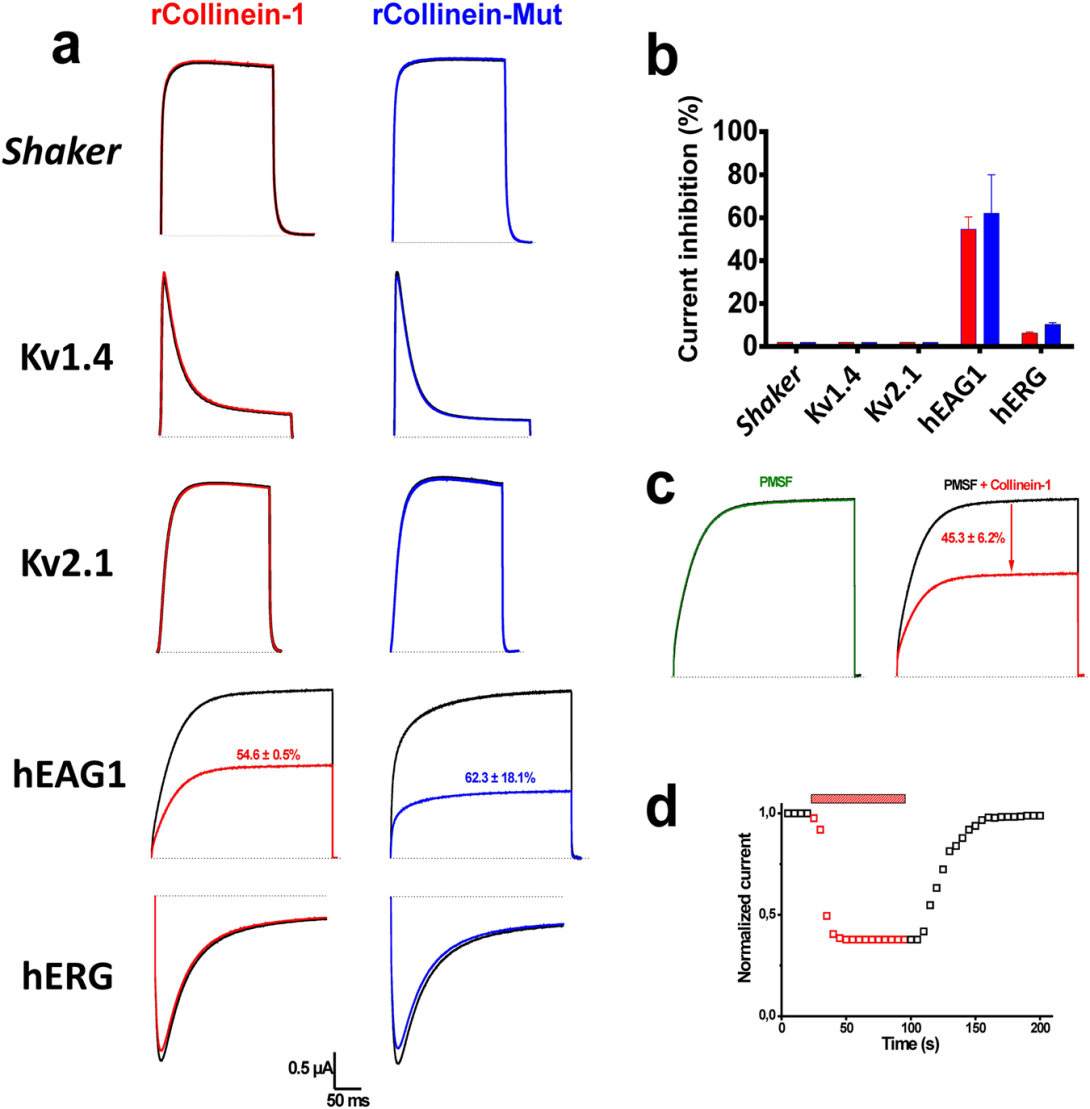
Electrophysiological characterization of recombinant and mutant (His43Arg) collinein-1 on Kv channels. (**a**) Selectivity screening of rCollinein-1 and rCollinein-mut on a panel of Kv channel isoforms. Current traces of a representative experiment are shown before (black) and after application of the samples (red and blue). Dotted line represents zero current. (**b**) Current inhibition (%) observed after addition of 5 µM rCollinein-1 (red) or rCollinein-mut (blue) in different Kv channel isoforms. Values are shown as means (±SEM) of 3 independent experiments (n = 9). (*) indicates significant differences (p < 0.0001). (**c**) Effect of the chemical serine protease inhibitor PMSF alone (green) and rCollinein-1 inhibited with PMSF (red) on evoked hEAG1 current. (**d**) Reversible inhibitory effect of rCollinein-1 on normalized current recorded as a function of time. rCollinein-1 takes 50 seconds to reach the maximum current blockade, with subsequent reversibility of the inhibitory effect after removal of the protein from the medium.

Collinein-1 is a 29.5 kDa thrombin-like serine protease isoform from *C. d. collilineatus* venom that cleaves preferentially the Aα chain of fibrinogen^23^. The recombinant form of collinein-1 (rCollinein-1) was previously produced with functional integrity, using the *Pichia pastoris* heterologous expression system^23,24^. The screening of collinein-1 activity on voltage-gated ion channels was performed with rCollinein-1 (5 μM) on different isoforms of voltage-gated potassium (Kv) and sodium (Nav) channels expressed in *Xenopus laevis* oocytes. Recombinant collinein-1 significantly inhibited the current evoked by hEAG1, and by a hairbreadth also the current through hERG1 (Kv11.1, *KCNH2*), but showed no effect on the A-type current elicited by the Kv1.4 channel, and on the delayed rectifier channels *Shaker* and Kv2.1 (Fig. 1a,b). This toxin was also unable to modulate the elicited currents of Nav (isoforms 1.1, 1.2, 1.3, 1.4, 1.5, 1.6, and 1.8) channels (Fig. S1).

The mutant form (His43 → Arg43) of collinein-1, named rCollinein-mut, was designed based on naturally-occurring mutant SVSPs that lack catalytic activity^25^. Like rCollinein-1, rCollinein-mut was produced using the *P. pastoris* system, and the absence of enzymatic activity was confirmed (Fig. S2). The electrophysiological characterization revealed rCollinein-mut blocked hEAG1 with similar efficiency as rCollinein-1, and the low blocking effect on hERG1 is unchanged. Most importantly, this experiment confirmed that the channel blocking effect does not depend on the catalytic activity of this SVTLE.

Collinein-1 blocked hEAG1 in a time and dose-dependent manner, with an IC_50_-value of 4.2 ± 0.5 μM for native collinein-1, 2.5 ± 0.3 μM for rCollinein-1, and 4.3 ± 0.8 μM for rCollinein-mut (Fig. S3A–C, respectively). hEAG1 current was tested at different voltages before and after treatment with the IC_50_ of native, recombinant, and mutant collinein-1. All tested proteins slightly shifted the V_1/2_ (voltage at which 50% of channels are activated) of hEAG1 (Fig. S3D–F, respectively) in the positive direction, demonstrating that these proteins may modulate the voltage-dependence of channel opening. However, this discrete modulatory effect indicates that the interaction of collinein-1 with the voltage-sensing domain has little contribution in channel inhibition, which is probably mainly induced by a physical obstruction of the pore (see molecular model further). In addition, we observed that all forms of collinein-1 blocked hEAG1 current more efficiently at negative potentials, with a decreasing efficiency as the potential increases, demonstrating a voltage-dependence for the binding of collinein-1 to hEAG1 (Fig. S3G–I). This result may indicate that collinein-1 shows a preference in interacting with hEAG1 in its closed state, since the inhibitory effect of the toxin is decreased at more depolarized potentials.

SVSPs present a highly conserved catalytic domain composed by the triad His57, Asp102, and Ser195^26^. The enzymatic activity of SVSPs is inhibited by a variety of natural and synthetic inhibitors^27^, particularly those that modify the reactive serine, such as phenylmethylsulfonyl fluoride (PMSF), which forms a covalent bond with this residue^28^. After treatment of rCollinein-1 with the chemical inhibitor PMSF, the collinein-PMSF complex blocked hEAG1 with the same efficiency as the non-inhibited enzyme. PMSF itself did not modify hEAG1 currents (Fig. 1c). Collinein-1 inhibits the hEAG1 channel in a rapid and reversible way, taking about 50 seconds to reach the maximum blockade effect (Fig. 1d).

The *ether-a-go-go* voltage-gated potassium channel family comprises the subfamilies EAG (Kv10), EAG-related gene (ERG; Kv11) and EAG-like (ELK; Kv12) K^+^ channels^29^. The first and most studied toxin that inhibits potassium channels from EAG family is ergtoxin-1 (ErgTx1) from *Centruroides elegans elegans* scorpion venom, which belongs to the γ-KTx subfamily and acts as a specific hERG1 blocker^30^. To date, several other toxins from scorpion, sea anemone and spider venoms that block or modulate channels from the EAG family are described in the literature. These peptides act on these channels by two main mechanisms: (i) by binding to the N-terminal part that delineates the entrance of the pore and in the transmembrane domains S5 and S6, promoting the physical occlusion of the ion-conducting pore or (ii) by interacting with regions of the voltage-sensing domain, thereby affecting the energy involved in channel opening and closing^31^. On the other hand, few animal toxins were reported to block or modulate channels from the EAG subfamily, such as the scorpion toxin κ-Hefutoxin-1^32^ and the sea anemone toxin APETx4^33^. In this study, we report for the first time a SVSP that selectively inhibits the activity of hEAG1 channel isoform.

## Collinein-1 putative Kv inhibitory motif is associated with the R79 residue that directly interacts to hEAG1 channel selectivity filter

The cryo-EM structure of rat ortholog EAG1 (rEAG1) revealed a shorter five-residue linker between S4 and S5 subunits that, differently from other Kv channel families, results in a non-domain swapped architecture of S1 to S6^34^. This unique EAG structure suggests a different mechanism of voltage-dependent gating for this channel family, which may be related to the entering of S4 to the cytoplasm in a down or hyperpolarized state, inducing a rotation in the C-linker and S6 that leads to a narrowing of the helical bundle to close the channel^34,35^. Another remarkable difference revealed by the rEAG1 structure is a 40-amino acid turret between S5 and the pore helix that extends ~25 Å outward from the membrane, surrounding the pore opening and preventing the binding of inhibitory toxins^34^. As we report here the first SVSP that blocks hEAG1, we were intrigued to know if other SVSPs can also bind to the extracellular 40-amino acid turret, being able to overcome this structural barrier.

To clarify the molecular mechanisms by which collinein-1 interacts with hEAG1, we performed an *in silico* molecular docking of the toxin on the ion channel. To cover the structural flexibility of SVSPs observed in loops related to substrate specificity^26^ (residues 20–26, 76–84 and 156–163, shown in magenta in Fig. S4), 14 homology models of collinein-1 were selected. Their docking was simulated to the extracellular domains of the cryoEM structure of the rEAG1 channel^34^ (Fig. S5). The stability of three solutions were validated by molecular dynamic simulation (Fig. S6) and selected for intermolecular interaction analysis. We have carefully chosen the first docking solution (Fig. S6A) as a possible model of interaction due to the toxin’s coupling to the potassium selectivity filter of hEAG1 channel.

**Figure 2:**
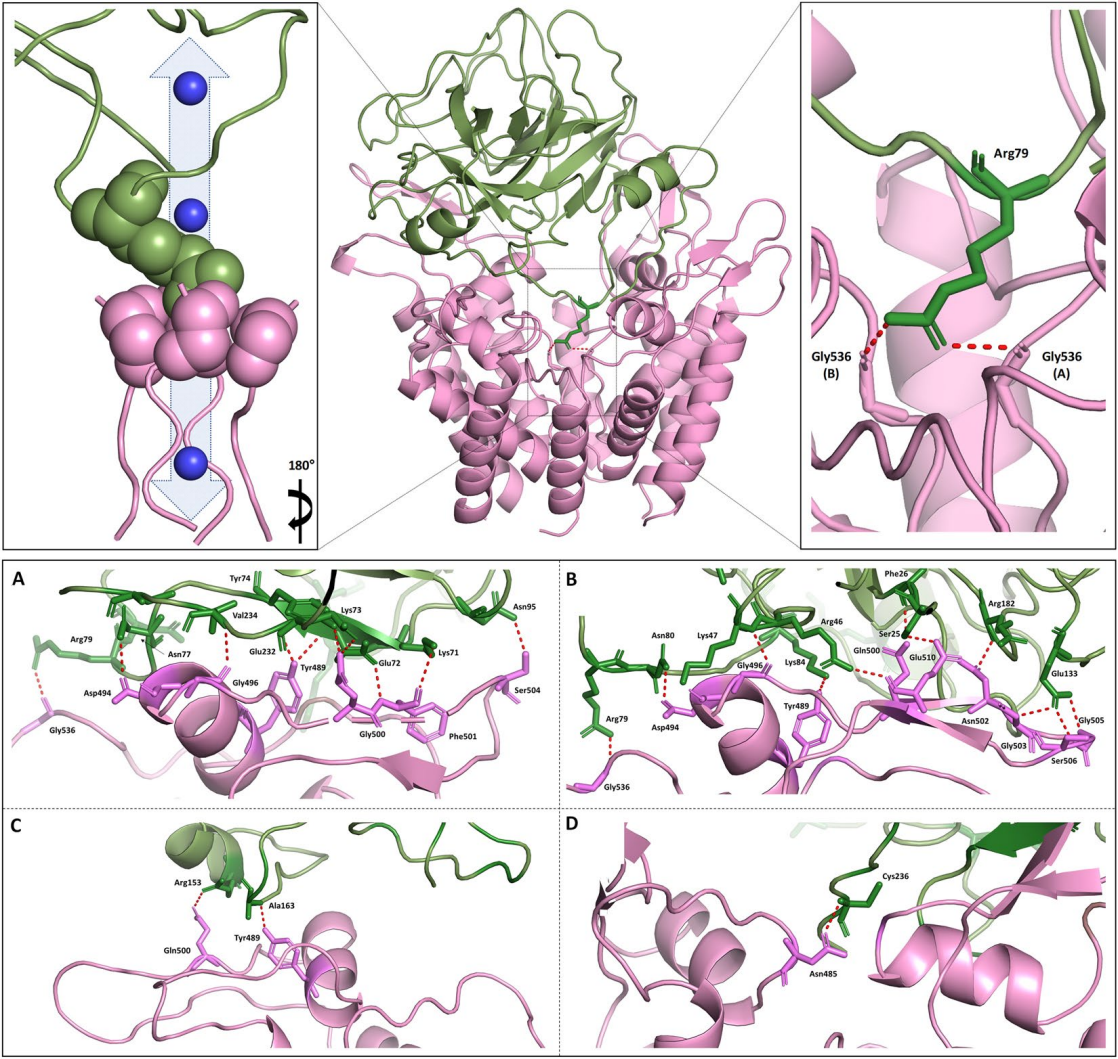
Selected docking solution for collinein-1 and hEAG1 channel. Side view of the docking solution validated by molecular dynamics. Left zoomed panel shows the blockage of hEAG1 conducting pore by collinein-1 Arg79 residue. Right zoomed panel shows the hydrogen bonds between the collinein-1 Arg79 residue and the two glycine 536 residues of the selectivity filter of two channel subunits (**A,B**). Interaction interfaces between collinein-1 and each channel’s subunits are shown in A–D panels, respectively. Collinein-1 is represented as a green cartoon and hEAG1 channel is represented in pink. The potassium ion is represented as a blue ball. Red dotted lines correspond to hydrogen bonds. *Channel subunits were aleatorily designated **A–D** for didactic purposes.

Based on the selected docking simulation, the molecular mechanism by which collinein-1 binds to hEAG1 involves numerous interactions. A total of 20 residues of collinein-1 make hydrogen bonds with 23 residues of the channel subunits, revealing a strong interaction interface between these two structures. Moreover, the docking solution revealed 17 hydrophobic contacts in the toxin and 24 in hEAG1 channel (Fig. S7). The solution revealed a putative pharmacophore that comprises an arginine residue (R79), whose side chain makes hydrogen bonds to the residues G536 from the potassium selectivity filter of the channel’s subunits A and B (where A → D denote the four subunits of the functional hEAG1 channel)^34^, perfectly fitting in the pore inlet (Fig. 2). The R79 protrudes from the serine protease 99-loop, which lines the edges of the enzyme active cleft. This loop acts like a “lid”, anchoring the channel’s vestibule by two additional hydrogen bonds formed by the toxin residues K84 and N80 in this loop, with Y489 and D494, respectively, from channel’s B subunit. This theoretical pore-blocking mechanism of collinein-1 is in accordance with the experimental electrophysiological characterization of the toxin, in which the toxin blocks hEAG1 channel but does not expressively shift the activation curve towards more positive potentials.

Figure S8 shows the interaction of collinein-1 with hEAG1, both top and bottom view, highlighting the blockage of the selectivity filter of hEAG1 channels by the R79 residue from collinein-1. Preceding the 99-loop, residues Y74 and N77 form a knot which form hydrogen bonds to Q500 and D494, respectively, from the channel’s A subunit. Altogether, these connecting points form a turn that attaches to the pore vestibule, precluding the potassium permeability. Associated to this turn, 15 other amino acids (Table S1), distributed throughout the toxin interaction interface, also contribute to the anchoring of collinein-1 to the channel’s four subunits (Fig. 2). According to the docking model, collinein-1 interacts mainly with the ion turret that is characteristic of EAG channels. As shown in Fig. S9, 12 amino acids from this turret make hydrogen bonds with collinein-1 and eight residues are involved in hydrophobic contacts with the toxin in this part of the channel. Among these residues, only one is conserved in all potassium channels used in the alignment (Fig. S9) and five residues are conserved only among ion channels from the EAG family. This observation may explain the selectivity of collinein-1 to the hEAG1 channel. Beyond interacting with the channel turrets, collinein-1 also makes interaction with three highly conserved residues from the selective filter (Fig. S9).

**Figure 3:**
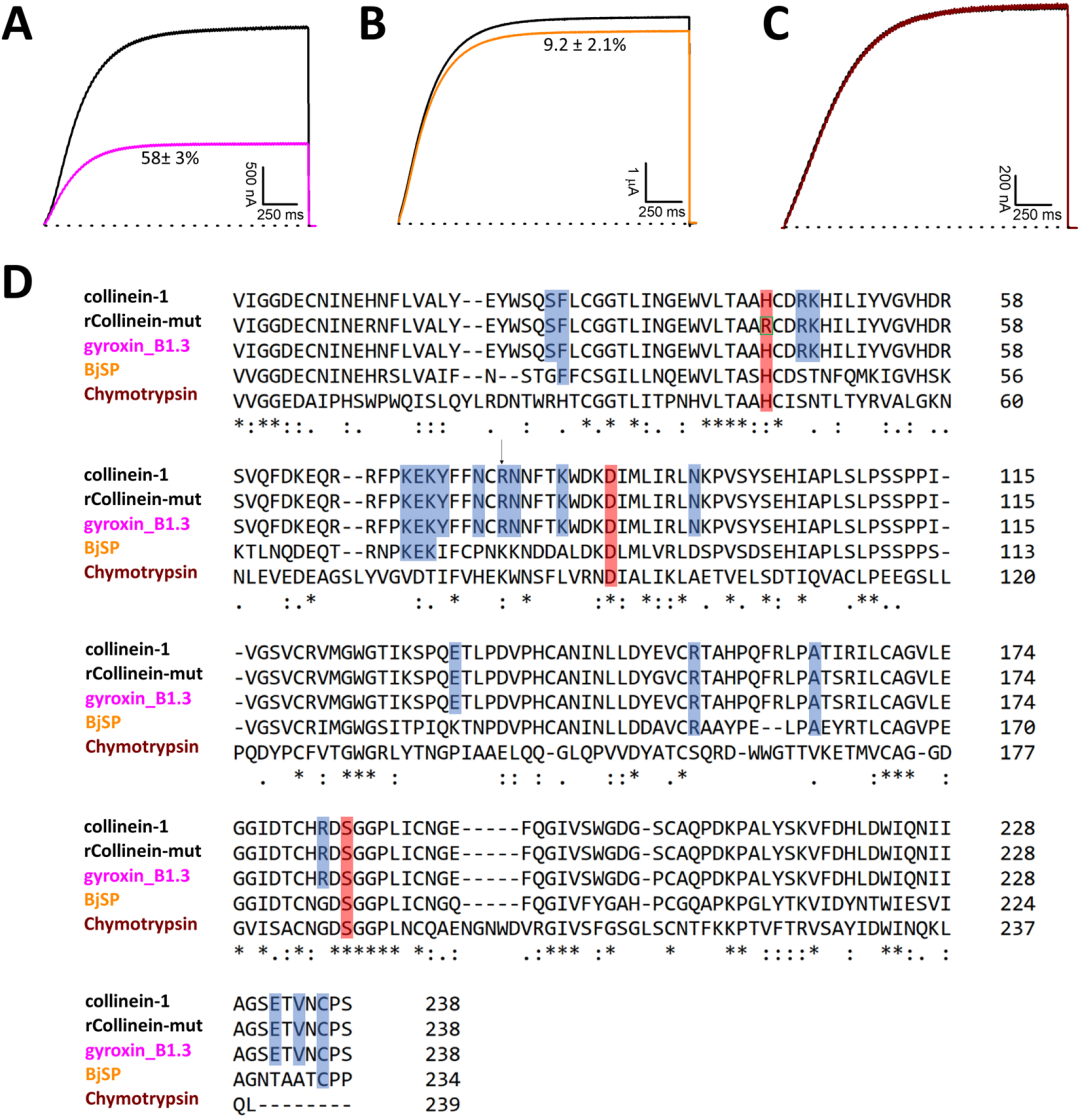
Electrophysiological profile of gyroxin_B1.3 (**A**), BjSP (**B**) and chymotrypsin (**C**), on hEAG1. Superimposed current traces before (black) and after application (purple, orange and brown traces) of the samples, at 5 µM. (**D**) Multiple alignment of the sequences of proteins tested on hEAG1. Universal Protein Resource accession code: A0A0S4FKT4 for collinein-1, B0FXM1 for gyroxin_B1.3, Q5W959 for BjSP and Q7M3E1 for chymotrypsin. Marked light blue residues represent the common interaction sites between the ligands and the target, according to the proposed docking model. Marked red residues represent the common catalytic triad of serine proteases. The green box highlights the mutation made (His43 → Arg43) on the sequence of collinein-1, to turn it catalytically inactive (rCollinein-mut). The arrow indicates the Arg79 residue.

The effects of gyroxin_B1.3, a SVSP from *C. d. terrificus* venom^36^, BjSP, a SVSP from *Bothrops jararaca* venom^37^, as well as the commercially available bovine chymotrypsin, were also evaluated on hEAG1. Gyroxin_B1.3, BjSP and bovine chymotrypsin display identities of 99%, 61% and 30% with collinein-1, respectively. Gyroxin_B1.3 shares all 20 residues from collinein-1 that interact with hEAG1, according to the proposed docking model, while BjSP shares seven conserved residues (Phe26, Lys71, Glu72, Lys73, Arg153, Ala163 and Cys236), and bovine chymotrypsin does not share any of them (Fig. 3, panel D, Table S1). Gyroxin_B1.3 inhibited 58 ± 3.0% of hEAG1 current, while BjSP inhibited 9.2 ± 2.1%, and no inhibition was observed by bovine chymotrypsin (Fig. 3, panels A, B and C, respectively), at 5 µM. These results strongly support the selected docking solution (Fig. S6A) and highlight the R79 residue as playing a fundamental role in the interaction of collinein-1 with this specific ion channel. As such, we have found a commonality among some SVSPs in blocking a potassium channel with potential antitumor activity.

Kv channel blocking toxins were initially associated with the presence of a so-called “functional dyad”, identified in several unrelated toxins from different animal sources, such as scorpions, snakes, and anemones^33,38–45^. This dyad is formed by a lysine, whose side chain is positioned at the pore inlet, and a hydrophobic residue (aromatic and aliphatic), located 7 ± 1 Å apart from the lysine^40,46,47^. An alternative theoretical model suggested for Kv channel blocker toxins is the “basic ring”, in which positively charged residues form salt bridges with negatively charged residues of the channel α-subunits^48^. On the other hand, toxins known to selectively inhibit hERG channels seem to interact with the channel by different mechanisms. The blocking effect of ErgTx on hERG probably involves a hydrophobic binding site formed by S5-P and P-S6 linkers in the outer vestibule of this channel^30^, while CnErg1, from *Centruroides noxius* scorpion venom, possibly interacts with the turret region of hERG by a cluster formed by a hydrophobic and a hydrophilic amino acids^49^. Different to other short scorpion toxins, BeKm-1, from *Buthus eupeus*, interacts with hERG channels through four critical residues in its structure, located in the α-helix and the following loop, thereby providing its specificity towards hERG channel^50^.

Although the presence of these functional motifs is an indicative of ionic channel pore-blocking effect, several channel inhibitory toxins lacking this molecular signature have already been identified^51–53^, suggesting that the mechanisms by which toxins block Kv channels are multifaceted and may require more complex interface contacts^47^. Likewise, in this study we propose a completely novel and complex mechanism of Kv channel blockage, describing the first animal toxin possibly capable of overcoming the EAG channel turrets, which directly interacts to the channel selectivity filter, leading to potassium flux obstruction.

## Collinein-1 blocks hEAG1 with higher efficiency as compared to hERG1: potential application in cancer research and antitumor drug design

Several studies demonstrated the abnormal ectopic expression of the EAG channels superfamily (particularly EAG and ERG) in different tumor cells. These channels play a crucial role in cancer progression, being involved in cell volume control, proliferation, migration and apoptosis^54^. The hEAG1 aberrant expression in cancer cells has aroused interest in the potential application of these channels as tumor markers, prognostic factor or therapeutic targets for antitumor drug discovery and design^55^.

Human EAG and ERG channels belong to the same Kv family and share about 47% homology in their primary structures^29^. Thus, most of EAG-channel blocker agents also lead to the inhibition of hERG channels, which can generate serious side effects, such as cardiac arrhythmia, long QT syndrome and sudden death^56,57^. In this context, it becomes evident that the discovery of new specific inhibitors for hEAG1 channels can lead to controlling cell proliferation and, consequently, diminish progression of some cancer cell lines. Moreover, these drugs can be used in combination with chemotherapeutic agents or be employed in chemoresistant disease to increase the patient survival^5^. The most studied inhibitors of hEAG1 are imipramine and astemizole, which present IC_50_ of 1.8 μM and 196 nM, respectively^58^. Tetrandrine, from traditional Chinese medicine, was recently reported to inhibit hEAG1 channel with an IC_50_ of 69.97 μM^59^. Although collinein-1 exhibits an IC_50_ greater than astemizole, it blocks the hEAG1 current with imipramine-comparable efficiency and with greater efficacy as compared to tetrandrine, which reinforces the importance of this molecule in blocking this oncogenic channel.

The screening of the effect of native, recombinant, and mutant collinein-1 on different Kvs revealed that these proteins inhibit both hEAG1 and hERG1 but showed a considerably greater blocking effect on hEAG1. Therefore, although collinein-1 is not a selective hEAG1 blocker, this protein inhibits the channel with higher efficiency when compared to hERG, being a molecule of interest for the development of a safer antitumor therapy.

**Figure 4:**
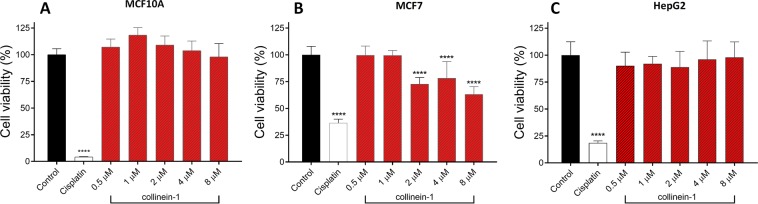
MTT-assessed cell viability of MCF10A (**A**), MCF7 (**B**) and HepG2 (**C**) after treatment with different concentrations of collinein-1. Results expressed by the mean ± SD for three independent experiments (n = 3). Negative control: PBS; Cisplatin was used as positive control. **p < 0.01 and ****p < 0.0001 in relation to the negative control (ANOVA, followed by the Tukey test).

In view of the antitumor potential of collinein-1, the cytotoxic dose-response effect of the toxin on two tumor cell lines (human hepatocellular carcinoma cell line HepG2 and human breast adenocarcinoma cell line MCF7) and on a non-tumorigenic human breast epithelial cell line (MCF10A) was assessed by the MTT (3-(4,5-Dimethylthiazol-2-yl)−2,5-diphenyltetrazolium bromide) assay (Fig. 4). Collinein-1 did not induce cytotoxicity at any of the concentrations tested in MCF10 (Fig. 4A) and HepG2 (Fig. 4C). On the other hand, in MCF7, the concentrations of 2 to 8 µM were effective in inducing cell death, although no dose-dependent effect was observed from the minimum effective concentration (Fig. 4B).

The overexpression of channels belonging to the EAG superfamily was demonstrated by RT-PCR and immunostaining in about 80% of breast cancers^60^. In MCF7 tumor cell line, hEAG1 channels are involved in increased proliferation and cell cycle progression^61^. Conversely, expression of hEAG1 in hepatocellular carcinoma cells seems to be limited to the early stages of neoplastic development, although the precise role of hEAG1 channels on this tumor line remains to be elucidated^62^. Since collinein-1 was able to reduce the viability of MCF7 tumor cells rather than HepG2, it is possible to suggest that the antitumor effect of the toxin may be related to the inhibition of hEAG1 channels in this tumor line. However, the antitumor mechanisms of collinein-1, as well as its effects on proliferation, apoptosis and cell cycle, remain to be comprehensively investigated.

The mutant collinein-1 lacks its catalytic activity but maintains the inhibitory effect on hEAG1 with high efficiency. In this sense, this molecule represents a step forward in the development of drugs targeting the treatment of malignant tumors expressing high levels of hEAG1 channels, since this mutation may not generate undesirable side effects related to blood coagulation, such as *in vivo* hypofibrinogenemia and interactions with blood coagulation factors.

## Methods

### Venom and materials

*C. d. collilineatus* venom was extracted from specimens collected in the state of Goiás, Brazil. After extraction, the venom was dried under vacuum at room temperature and stored at −20 °C. The snakes were kept in captivity at the Serpentarium Bioagents of Batatais - SP, Brazil. *P. pastoris* KM71H strain, pPICZαA expression plasmid, and Zeocin™ were purchased from Invitrogen™ (Carlsbad, USA). The restriction enzyme used in plasmid linearization was purchased from New England Biolabs (Ipswich, USA). Other reagents not specified here were of analytical grade. Other materials and equipment used are described throughout the methods section.

### Purification of native collinein-1

Native collinein-1 was purified according to the method described by Boldrini-França *et al*.^23^.

### Production and purification of recombinant Collinein-1 (rCollinein-1)

Recombinant collinein-1 was produced as described by Boldrini-França *et al*.^24^*. Pichia pastoris* cells, transformed with the recombinant plasmid (containing collinein-1 coding sequence), was pre-inoculated with 10 mL of BMGY medium (1% yeast extract, 2% peptone, 1.34% YNB, 4 × 10^−5^ M biotin, 1% glycerol, 100 mM phosphate potassium pH 6.0) and incubated at 30 °C under constant stirring of 200 rpm. After 24 h, the culture was inoculated in 500 mL of BMGY medium and incubated in the same conditions until reaching an optical density of 2 to 6 at 600 nm. After achieving the desired optical density, the culture was centrifuged at 1500 × *g*, the supernatant was discarded, and the cells were resuspended in 100 mL of BMMY medium (1% yeast extract, 1.34% YNB, 4 × 10^−5^ M biotin, 1% methanol, 100 mM potassium phosphate pH 6.0) and incubated at 26 °C under constant stirring of 200 rpm. Methanol was replaced every 24 h at a final concentration of 0.75%, for the maintenance of induction. After 96 h of induction, the culture was centrifuged at 10,000 × *g* and the supernatant was separated and filtered. rCollinein-1 was purified from the culture supernatant by IMAC (Immobilized Metal Ion Affinity Chromatography) using a Ni^2+^-Agarose resin (NTA Agarose - Qiagen) and gravitational flow. Elution of the recombinant protein was performed with a gradient segmented to 250 mM imidazole. Fractions containing rCollinein-1 were dialyzed against ultrapure water, lyophilized and resuspended in 50 mM sodium acetate buffer, pH 5.0. The resuspended fraction was then applied in a cation exchange column (CMC-52, 20 cm × 4 cm) at a flow rate of 0.7 mL/min using 50 mM sodium acetate buffer, pH 5.0 as mobile phase. Elution was performed in a segmented gradient of the same buffer, up to 1 M, collecting 1.5 mL per tube. The absorbance was monitored at 280 nm. The fractions eluted from the column were analyzed by 13.5% SDS-PAGE, dialyzed against ultrapure water, lyophilized and stored at −20 °C until use.

### Expression and purification of mutant Collinein-1 (rCollinein-mut)

rCollinein-mut coding gene was synthesized and cloned into *Xho*I and *No*tI restriction sites of pPICZαA vector by GenScript^®^ (New Jersey, USA). The recombinant vector was linearized with the restriction enzyme *Pme*I and used to transform *P. pastoris* cells (KM71H strain) by electroporation in a GenePulser II (Bio-Rad Laboratories, Hercules, USA) at 1500 V, 25 mF, and 200 Ω. After transformation, cells were plated in YPDS agar medium (1% yeast extract, 2% peptone, 2% dextrose, 1 M sorbitol, 2% agar) containing 100 mg/mL of Zeocin and incubated at 30 °C for 3–5 days for selection of transformants. For the selection of recombinant colonies containing multiple copies of the gene, cells (200 μL) were plated on YPDS medium containing 500 mg/mL Zeocin and incubated under the conditions described above. The recombinant colonies were subjected to colony PCR, and the product was visualized on a 1% agarose gel to confirm that the insert was incorporated in the yeast genomic DNA. Expression and purification of rCollinein-mut was performed in the same conditions described for rCollinein-1.

### Catalytic activity upon the chromogenic substrate S-2302

The catalytic activity of the native collinein-1 and rCollinein-mut was evaluated with the chromogenic substrate S-2302 by Chromogenix^®^ (Bedford, MA, USA) for plasma kallikrein (H-D-Pro-Phe-Arg-pNA•2HCl). Substrate hydrolysis was determined by incubating different doses of the proteins (1,25, 2,5, 5, and 10 μg) with 0.4 mM of substrates in Tris-HCl 0.05 M, pH 7.5, containing 0.05 M CaCl_2_ for 40 min at 37 °C. Substrate hydrolysis was spectrophotometrically monitored at 405 nm using Versa Max Microplate reader (Molecular Devices, Sunnyvale, USA). Assays were performed in a series of three replicates, and the data were fitted with standard errors using GraphPad Prism software, version 5.0 (GraphPad Software, San Diego, USA). Statistical analysis of the results was performed using the Student’s *t* test or one-way ANOVA method with Tukey’s post-test, comparing all treatments to the negative controls and considering values of *p* < 0.05 as significant.

### Expression of voltage-gated ion channels in *Xenopus* oocytes

For expression of Kv (rKv1.4, Shaker IR, rKv2.1, hERG, hEAG1), and Nav (rNav1.1, rNav1.2, rNav1.3, rNav1.4, hNav1.5, mNav1.6, hNav1.8) channels, as well as the auxiliary Nav subunits rβ1 and hβ1, in *Xenopus laevis* oocytes, the linearized plasmids were transcribed using the T7 or SP6 mMESSAGEmMACHINE transcription kit (Ambion, Austin, TX, USA). Stage V-VI *X. laevis* (African clawed frog) oocytes were isolated by partial ovariectomy. Mature female animals were purchased from Nasco (Fort Atkinson, Wisconsin, USA) and were housed in the Aquatic Facility (KU Leuven) in compliance with the regulations of the European Union (EU) concerning the welfare of laboratory animals as declared in Directive 2010/63/EU. The use of *X*. *laevis* was approved by the Animal Ethics Committee of the KU Leuven with the license number LA1210239. Prior to harvesting the oocytes, the animals were anesthetized by a 15-min submersion in 0.1% tricaine methanesulfonate (pH 7.0). Isolated oocytes were defolliculated with 1.5 mg/mL collagenase. Oocyte microinjection was performed using a microinjector (Drummond Scientifc^®^, USA), with a programmed injection volume of 4–50 nL of cRNA. The oocytes were incubated in ND96 solution (96 mM NaCl; 2 mM KCl; 1.8 mM CaCl_2_; 2 mM MgCl_2_ and 5 mM HEPES, pH 7.4), supplemented with 50 mg/L gentamicin sulfate.

### Electrophysiological measures

The electrophysiological measurements were performed by the voltage-clamp technique with two microelectrodes. Data were obtained using a GeneClamp 500 amplifier (Axon Instruments^®^, USA), connected to a computer equipped with a Windows XP operating system (Microsoft^®^, USA) and Camplex9 software (Axon Instruments^®^, USA), enabling data acquisition and storage. Glass micropipettes were produced using glass capillaries (borosilicate WPI 1B120-6) and drawn in a WPI (Word Precision Instruments^®^) manual stretcher. The bath and perfusion solution consisted of ND96 solution.

Whole-cell currents of oocytes were recorded 1 to 5 days after injection. Currents and voltage electrodes were filled with 3 M KCl and their resistance were adjusted from 0.7 to 1.0 MΩ. Kv currents were sampled at 1 kHz and filtered at 500 Hz, using a four-pole low-pass Bessel filter, except for Kv10.1 and hERG, which currents were filtered at 1 kHz. Leak subtraction was performed using a P/4 protocol. Kv channel currents (except hERG, hEAG1 and Kv2.1) were evoked by 500 ms depolarizations to 0 mV followed by a 500 ms pulse to −50 mV, from a holding potential of −90 mV. Current traces of hERG channels were elicited by applying a +40 mV prepulse for 2 s followed by a step of −120 mV for 2 s. Kv2.1 current was elicited by 500 ms pulses to +20 mV from a holding potential of −90 mV. To determine the current-voltage relationship, hEAG current traces were evoked by a series of depolarization steps from −90 to +65 mV (ΔV = 5 mV) from a holding potential of 90 mV.

Nav currents were sampled at 20 kHz by using a 4-pole low-pass Bessel filter. Both currents were filtered at 1 kHz. Leak subtraction was performed by using a P/4 protocol. For the electrophysiological analyses, the protocol for Nav channels was applied from a holding potential of −90 mV, with a start-to-start interval of 0.2 Hz. Sodium current traces were evoked by a 100 ms depolarization to 0 mV, in ND-96 solution.

To calculate IC50, a concentration-response curve was constructed and fitted with the logistic dose-response equation, $y=\frac{A1-A2}{1+{(IC50/[toxin])}^{nH}}+A2$ where y represents the percentage of current inhibition, A1 the initial inhibition at the lowest toxin concentration (0%), A2 the final inhibition at the highest toxin concentration, IC50 the half maximal inhibitory toxin concentration and *n*H the Hill coefficient.

For the assays, different concentrations of the toxins previously diluted in ND96 were used. Next, this solution was added to the oocyte-containing chamber and immediately homogenized, thereby obtaining the desired final concentration. To assess the role of enzymatic activity of the toxins in channel inhibition, rCollinein-1 was treated with 10 mM of the chemical inhibitor phenylmethylsulfonyl fluoride (PMSF) for 30 min at room temperature and its activity on hEAG1 was tested.

### Homology modeling and protein-protein docking

The homology model of collinein-1 was built using MODELLER 9.13^63^ and the following structures as templates: i) AhV_TL-I, a glycosylated snake venom thrombin-like enzyme from *Agkistrodon halys* (PDB id: 4E7N; 68% identity); ii) native protein C activator from *Agkistrodon contortrix contortrix* venom^64^ (PDB id: 2AIP; 66% identity); iii) Plasminogen activator (TSV-PA) from *Trimeresurus stejnegeri* venom^26^ (PDB id: 1BQY; 61% identity); iv) saxthrombin from *Gloydius saxatilis* venom^65^ (PDB id: 3S69; 60% identity); v) Russell’s viper venom serine proteinase from *Daboia siamensis* venom^66^, RVV-V (closed-form) (PDB id: 3S9A; 58% identity); vi) all listed models together. The template structures were chosen based on resolution, model quality and structural similarity. For each template, five models of collinein-1 were generated, totaling 35 models, from which 14 were selected for molecular docking analysis to reduce structural redundancy (Fig. S4). The use of different models figures up local flexibility and may improve the quality of the docking solutions.

The structure of the rat EAG channel was previously elucidated by cryoelectron microscopy^34^ (Kv10.1 cryoEM model, PDB id: 5K7L) and this structure was used to simulate the docking to human EAG, since their extra-cellular and transmembrane domains are identical. MODELLER 9.13^63^ was employed to model the five missing residues (numbered 407–411) in an extracellular loop by *ab initio*, constraining the modelling to be identical to each monomer. For each protein pair used in the molecular docking, 4,000 docking solutions were generated with ZDOCK (resulting in a total of 56,000 poses), using high rotational sampling density of 6° with different initial rotations for each test case to avoid bias^67^ and restricting docking site to extracellular region of the channel. The 20 top-ranked ZDOCK solutions were hydrogenated and refined with RosettaDock 2.0^68^ and reranked with ZRANK^69^. Since ZDOCK essentially considers rigid protein-protein interactions, excluding any possibility of structural rearrangement, 300 locally improved models were generated for each of the 20 top-ranked solutions using the Monte Carlo and local high-resolution refinement methods of RosettaDock. The solutions that presented a root-main-square deviation (RMSD) above 7 Å were excluded. The RMSDs were calculated using the distances of Cα from pair of proteases after receptor coordinates were superposed. The validation of the selected solutions was performed by Molecular Dynamics (MD) using GROMACS^70^. The contact surface of the theoretical complex and the amino acid residues interactions in the intermolecular interface were assessed with LigPlot^+^ version 1.4^71^. Docking solutions were visualized using PyMOL Molecular Graphics System, Version 2.0 Schrödinger, LLC.

### Cell cultures

Human breast adenocarcinoma (MCF7, HTB-22), non-tumorigenic human breast epithelial cell (MCF10A, CRL-10317) and human liver carcinoma (HepG2, HB-8065) cell lines were obtained from American Type Culture Collection (ATCC, Rockville, MD, USA). The MCF7 and MCF10A grown in RPMI 1640 medium supplemented with 10% fetal bovine serum, 1% streptomycin, and 1% penicillin. HepG2 cells grown in DMEM (Dulbecco’s Modified Eagle Medium), supplemented with 10% fetal bovine serum, 1% streptomycin, and 1% penicillin. Vials containing the cells were incubated (37 °C, humidified atmosphere, 5% CO_2_) until the cultures reached confluence (∼5 × 10^6^ cells), when a subculture was required.

### MTT-assessed cytotoxic effect on HepG2 and MCF7 tumor cells and non-tumor MCF10A

Cytotoxic effect of collinein-1 on HepG2, MCF7, and MCF10A was assessed by the MTT method^72^. Prior to the assays, cell viability was evaluated using Tripan blue in a ratio of 1:1. Aliquots of 10 μL of cell suspension were counted on a Countess^TM^ cell counting chamber slides (Invitrogen, Carlsbad, USA). Only cultures with viability greater than 98% were used for the experiments. The cytotoxicity test was performed in 96 well plates using 5 × 10^4^ cells/well, with a final volume of 100 μL. Cells were plated and incubated for 24 hours at 37 °C in humidified 5% CO_2_ atmosphere. After this, cells from HepG2, MCF10A and MCF7 tumor cell lines were treated with 50 μL of medium (negative control) or the same volume of medium containing 0.5–12 μM of collinein-1. Cisplatin (Incel-Darrow^®^) (final concentration of 250 μg/mL) was used as positive control. Cells were incubated again for 24 hours at 37 °C in humidified 5% CO_2_ atmosphere, for cytotoxicity evaluation. After 24 hours of treatment, 20 μL of tretrazolium 3-(4,5-dimethylthiazol-2-yl)2,5-diphenylbromide (MTT) (Sigma-Aldrich, San Luis, Missouri, EUA) were applied to the wells and the plates were incubated again for 3 hours in the same conditions. The plates were centrifuged for 5 min at 900 × *g* and then inverted for discard of the supernatant. DMSO (100 μL) (Sigma-Aldrich, San Luis, Missouri, EUA) was added in each well, and kept under stirring until the formazan crystals were completely dissolved. Plates were analyzed by absorbance at 570 nm and results were expressed as percentage of cell viability in comparison to the negative control. Three independent assays were performed in a series of three replicates, and the data were fitted with standard errors using GraphPad Prism software, version 5.0 (GraphPad Software, San Diego, USA). Statistical analysis of the results was performed using the Student’s *t* test or one-way ANOVA method with Tukey’s post-test, comparing all treatments to the negative controls and considering values of *p* < 0.05 as significant.

## Supplementary information

Supplementary Dataset 1.

## Data Availability

The datasets generated during and/or analyzed during the current study are available from the corresponding author on reasonable request.
