# Supplementary material for: Beyond hemostasis: a snake venom serine protease with potassium channel blocking and potential antitumor activities
Source: Sci Rep. 2020 Mar 11;10:4476. doi: 10.1038/s41598-020-61258-x (PMC7066243; doi:10.1038/s41598-020-61258-x)
Supplement: Supplementary file 1 — Supplementary Dataset 1. [file 41598_2020_61258_MOESM1_ESM.pdf]

SUPPLEMENTARY MATERIAL

Manuscript title: Beyond hemostasis: a snake venom serine protease with potassium channel blocking and potential antitumor activities

Authors: Johara Boldrini-França[1,5#], Ernesto Lopes Pinheiro-Junior[1,2#], Steve Peigneur[2], Manuela Berto Pucca[3], Felipe Augusto Cerni[1], Rafael Junqueira Borges[4], Tássia Rafaella Costa[1], Sante Emmanuel Imai Carone[1], Marcos Roberto de Mattos Fontes[4], Suely Vilela Sampaio[1], Eliane Candiani Arantes[1*] and Jan Tytgat[2*]

Affiliations:
[1]School of Pharmaceutical Sciences of Ribeirão Preto, University of São Paulo, Av. do Café s/nº, 14040-903, Ribeirão Preto, SP Brazil;
[2]Toxicology and Pharmacology, KU Leuven, O&N II Herestraat 49 - PO 922, 3000 Leuven, Belgium;
[3]Medical School of Roraima, Federal University of Roraima, Av. Capitão Ene Garcez, 2413, Bairro Aeroporto, 69310-970, Boa Vista, RR, Brazil;
[4]Institute of Biosciences, São Paulo State University (UNESP), Rua Prof. Dr. Antonio Celso Wagner Zanin, 250, 18618-689, Botucatu, SP, Brazil;
[5]University of Vila Velha, Av. Comissário José Dantas de Melo, 21, Boa Vista II, 29102-920, Vila Velha, ES, Brazil.

Corresponding authors: [*]Jan Tytgat, Toxicology and Pharmacology, University of Leuven (KU Leuven), Campus Gasthuisberg, O&N2, PO Box 922, Herestraat 49, 3000 Leuven, Belgium, phone: +32 16 323403, Fax: +32 16 323405, e-mail address: jan.tytgat@kuleuven.be
[*]Eliane Candiani Arantes, School of Pharmaceutical Sciences of Ribeirão Preto, University of São Paulo (USP), Av. do Café s/nº, Animal Toxins Laboratory, room 64A-A, Monte Alegre, 14040-903, Ribeirão Preto, SP, Brazil, phone: +55 16 33154275, Fax: +55 16 33154880, e-mail address: ecabraga@fcfrp.usp.br

#Johara Boldrini-França and Ernesto Lopes Pinheiro-Junior contributed equally to this work.

Fig. S1.

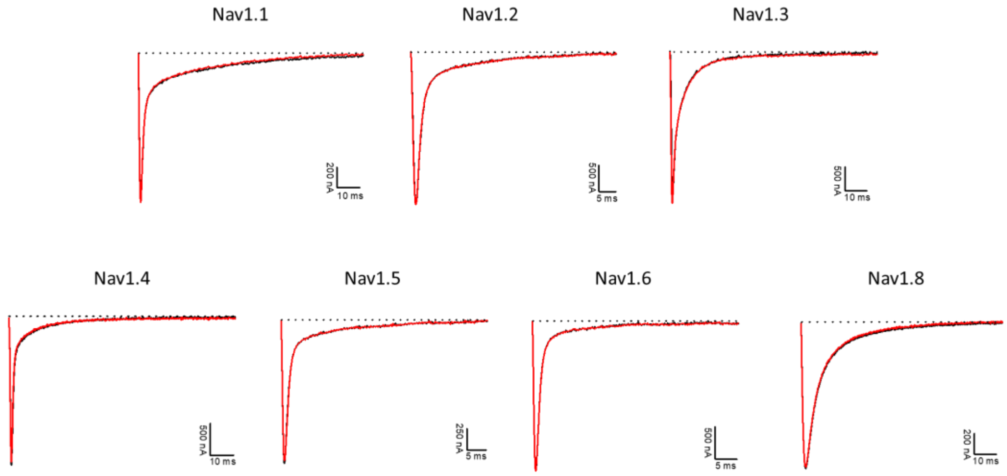

Figure S1. Electrophysiological characterization of rCollinein-1 on Nav channels. Selectivity screening of rCollinein-1 (5 µM) on a panel of Nav channel isoforms. Current traces of a representative experiment are shown before (black) and after application of the sample (red). Dotted line represents zero current.

Fig. S2.

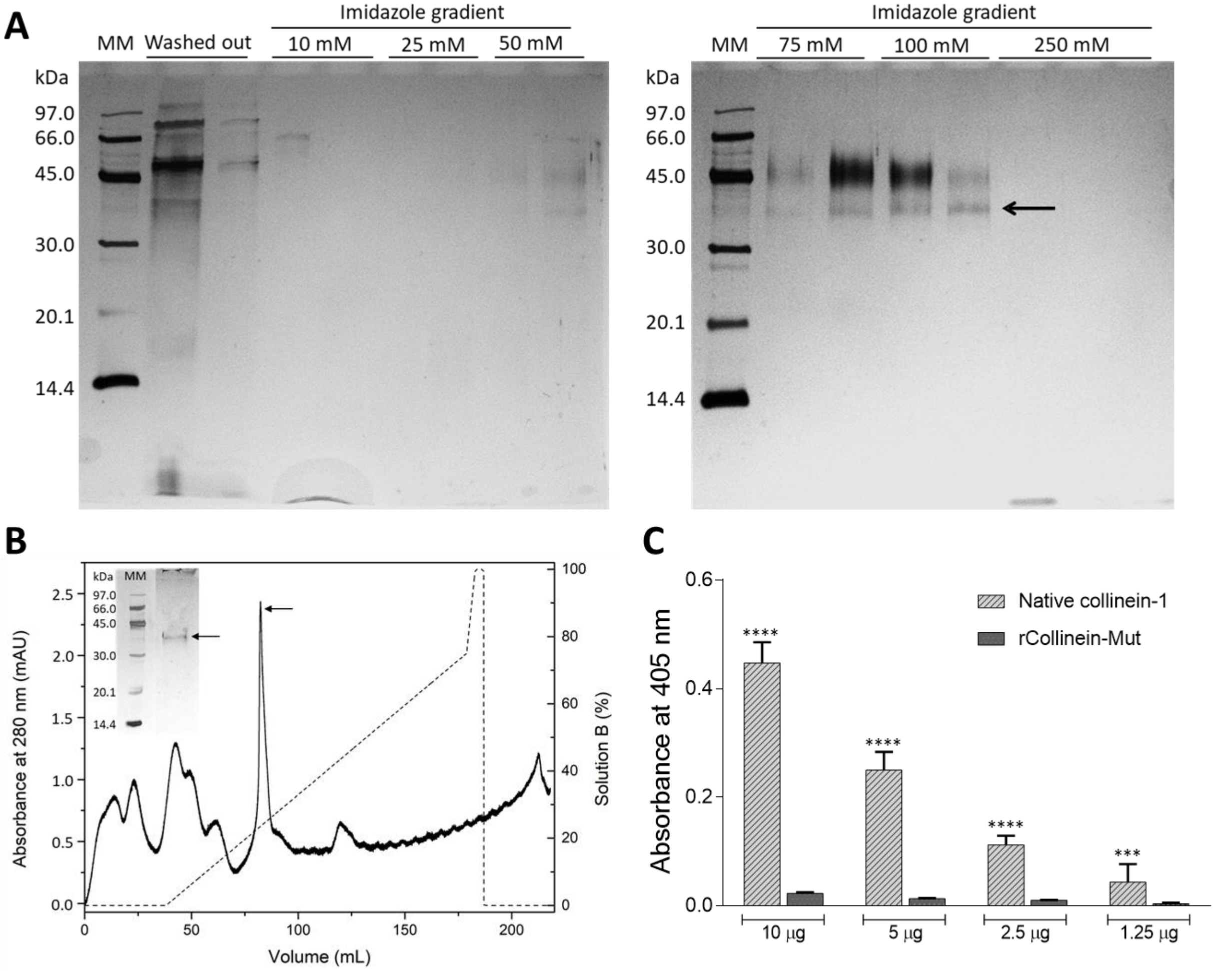

Figure S2. Expression and purification of rCollinein-mut. (A) Electrophoretic profile of fractions obtained in the first step of rCollinein-mut purification by IMAC using a Ni^{2+}-Agarose column. Elution of the recombinant protein was performed with a segmented gradient from 10 mM to 250 mM imidazole. MM, molecular marker; washed out, proteins eluted from the column with buffer lacking imidazole. **(B)** Chromatographic profile of the "100 mM imidazole fraction" in a cation exchange column CMC-52. Elution was performed in a segmented gradient of 50 mM sodium acetate buffer, pH 5.0, up to 1 M. The fractions eluted from the column were analyzed by 13.5% SDS-PAGE (inserted panel). The recombinant protein is indicated by the arrow. **(C)** Catalytic activity of native collinein-1 and rCollinein-mut on the chromogenic substrate for plasmatic kallikrein (S-2302). One activity unit was defined as the increase of 0.01 unit of absorbance at 405 nm. Results were expressed as mean ± SD (n = 3). (***) $p < 0.05$ relative to native protein; (****) $p < 0.001$ relative to native protein.

Fig. S3.

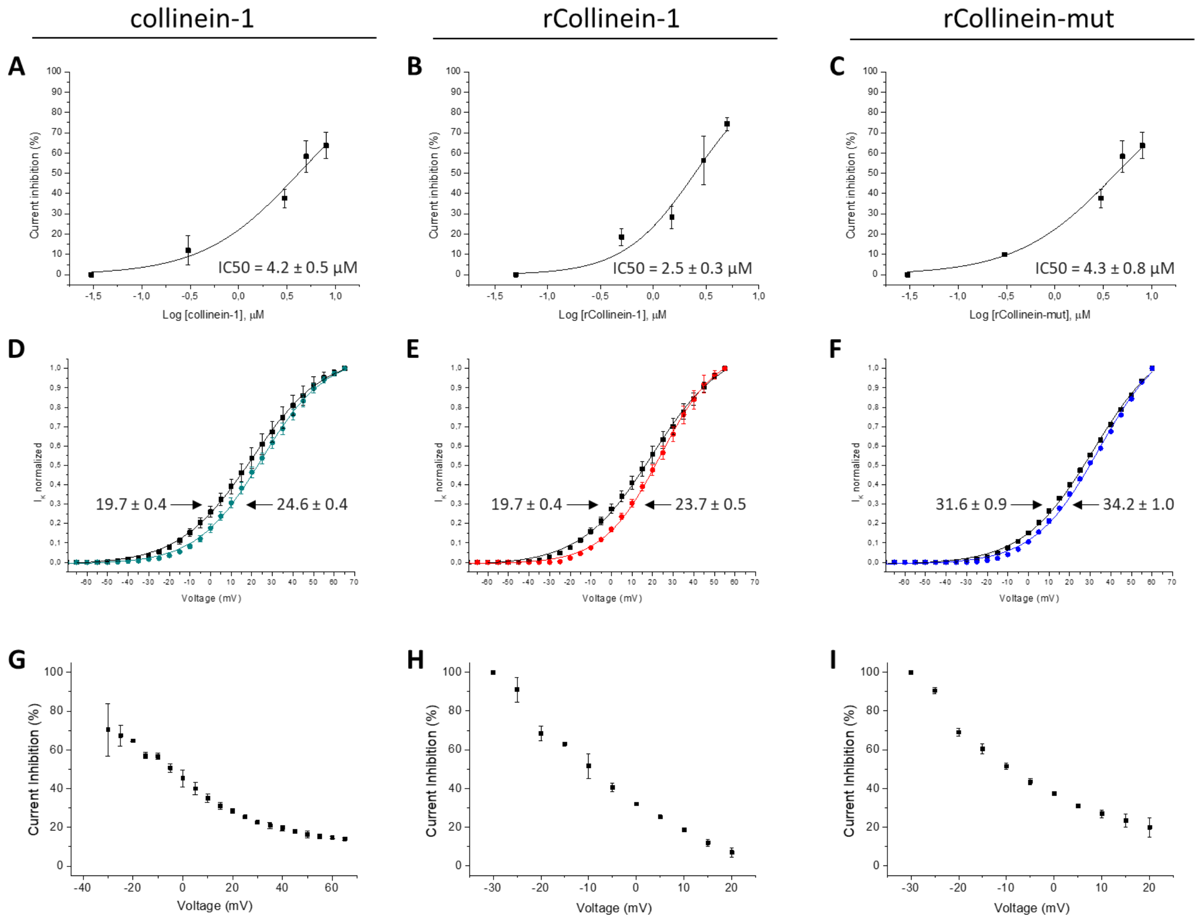

Figure S3. Concentration- and voltage-dependent effect of native, recombinant and mutant collinein-1 on evoked hEAG1 currents. (A, B and C) Concentration-dependency hEAG1 current inhibition of native, recombinant and mutant collinein-1, respectively. Plot fitted with a logistic equation shows the current inhibition (%) in function of toxin concentration. **(D, E and F)** Normalized currents of native (dark cyan), recombinant (red) and mutant collinein-1 (blue), respectively, elicited in ND-96 solution, were plotted versus the applied pulse potentials (mV) in control and toxin condition. The data points were fitted with the Boltzmann equation. **(G, H and I)** The inhibited current (%) observed after addition of native, recombinant and mutant collinein-1, respectively, plotted versus the applied pulse potential.

Fig. S4.

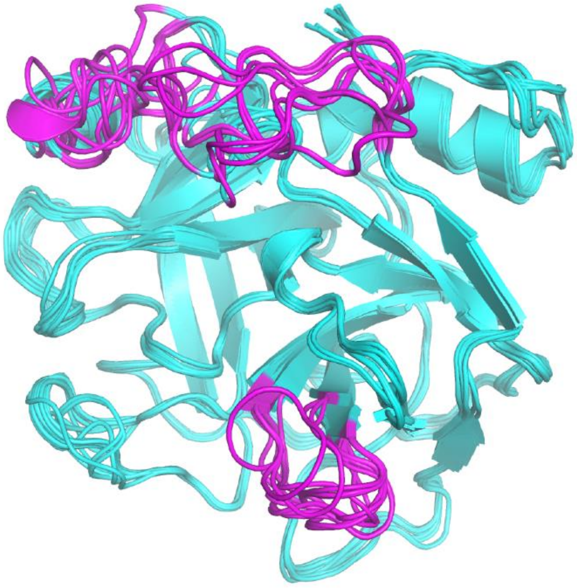

Figure S4. Collinein-1 structures selected from homology modelling for docking simulation. The selected structures used as templates for homology modelling of collinein-1 have a common fold but differ in the loops related to substrate specificity (residues 20-26, 76-84 and 156-163 shown in magenta). For each template, five collinein-1 models were generated, totaling 35 models. Only the shown 14 models were selected for docking simulation to reduce structural redundancy.

Fig. S5.

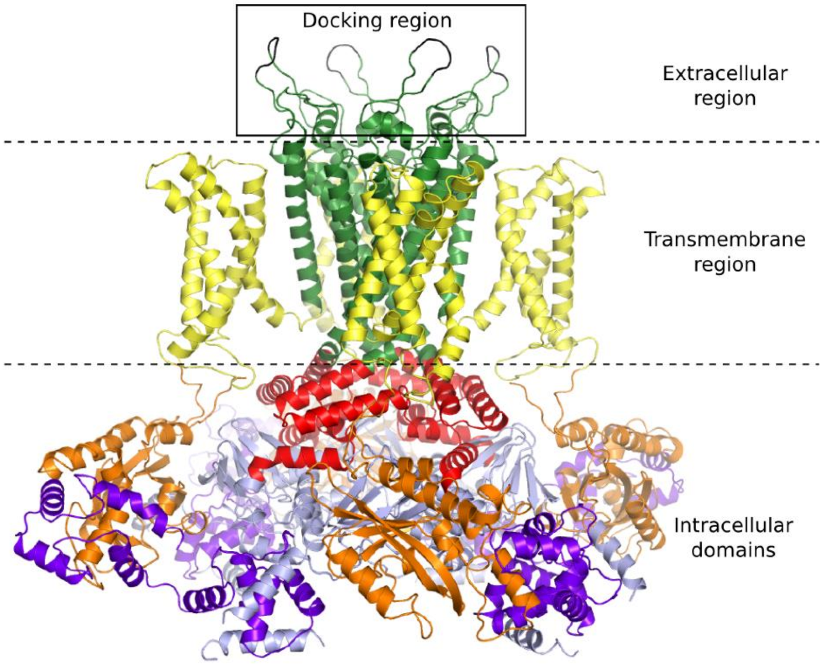

Figure S5. Molecular model of hEAG1 channel based on the Cryo-EM structure of the rEAG1 channel (PDB id: 5K7L). The program MODELLER was used to model the five missing residues (407-411 residues colored in black in an extracellular loop) by *ab initio* constraining the modelling to be identical to each monomer. Some intracellular domains were truncated to minimize the model. The molecular section used for docking simulation is highlighted in the black box.

Fig. S6.

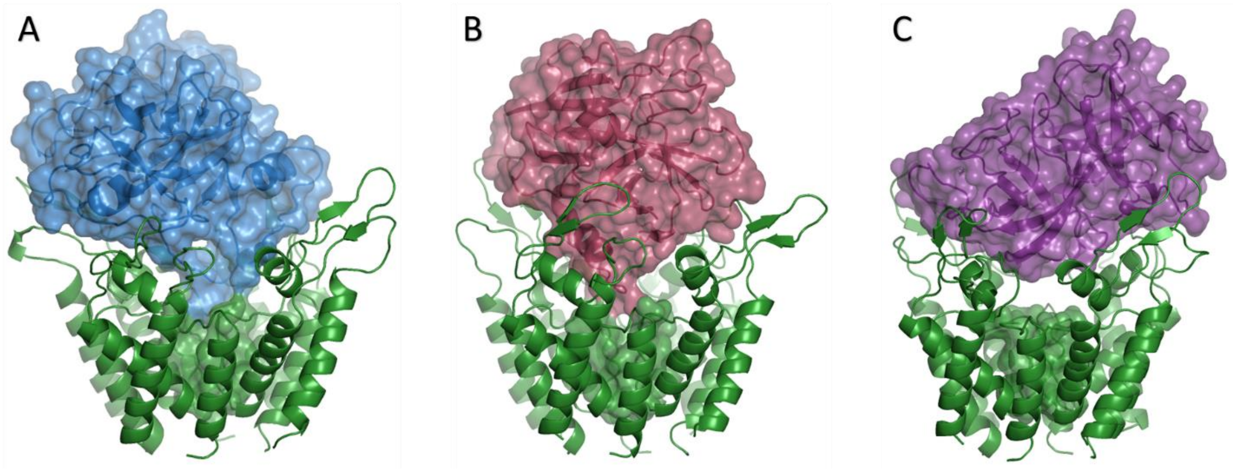

Figure S6. Top-ranked docking solutions for collinein-1 and hEAG1 channel interaction after validation and refinement by molecular dynamic simulations. The extracellular portion of the hEAG1 channel, with which the molecular docking was performed, is shown in green. The structures corresponding to collinein-1 are represented in blue **(A)**, red **(B)** and purple **(C)**. Model A was selected for intermolecular interaction analysis.

Fig. S7.

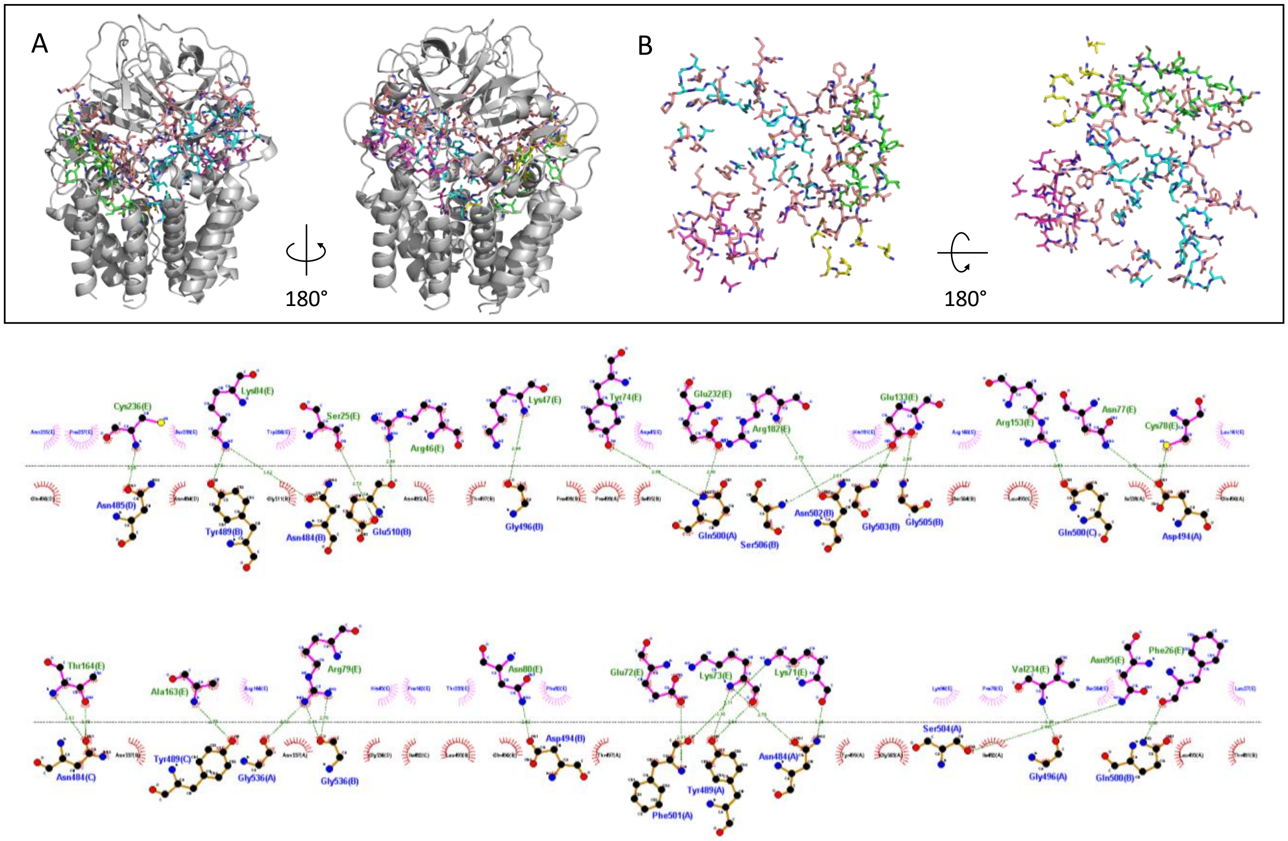

Figure S7. Molecular interactions between collinein-1 and hEAG1 channel structures. Upper panel: amino acid residues involved in the interaction interface are represented in colored sticks. Collinein-1 residues are represented in salmon, while channel residues are represented in green (subunit A), blue (subunit B), pink (subunit C) and yellow (subunit D). (A) Front and back view of the docking solution. (B) Up and down view of the interaction interface residues. Lower panel: hydrogen bonds and hydrophobic contacts between collinein-1 and hEAG1 channel. The amino acid side chains are shown in ball-and-stick representation. Hydrogen bounds are represented in green dotted lines, while the spoked arcs represent protein residues making hydrophobic contacts with the ligand. The amino acids above the dotted lines belong to collinein-1 and the amino acids below the dotted lines belong to hEAG1 subunits A, B, C and D (automatic designation performed by PyMol software).

Fig. S8.

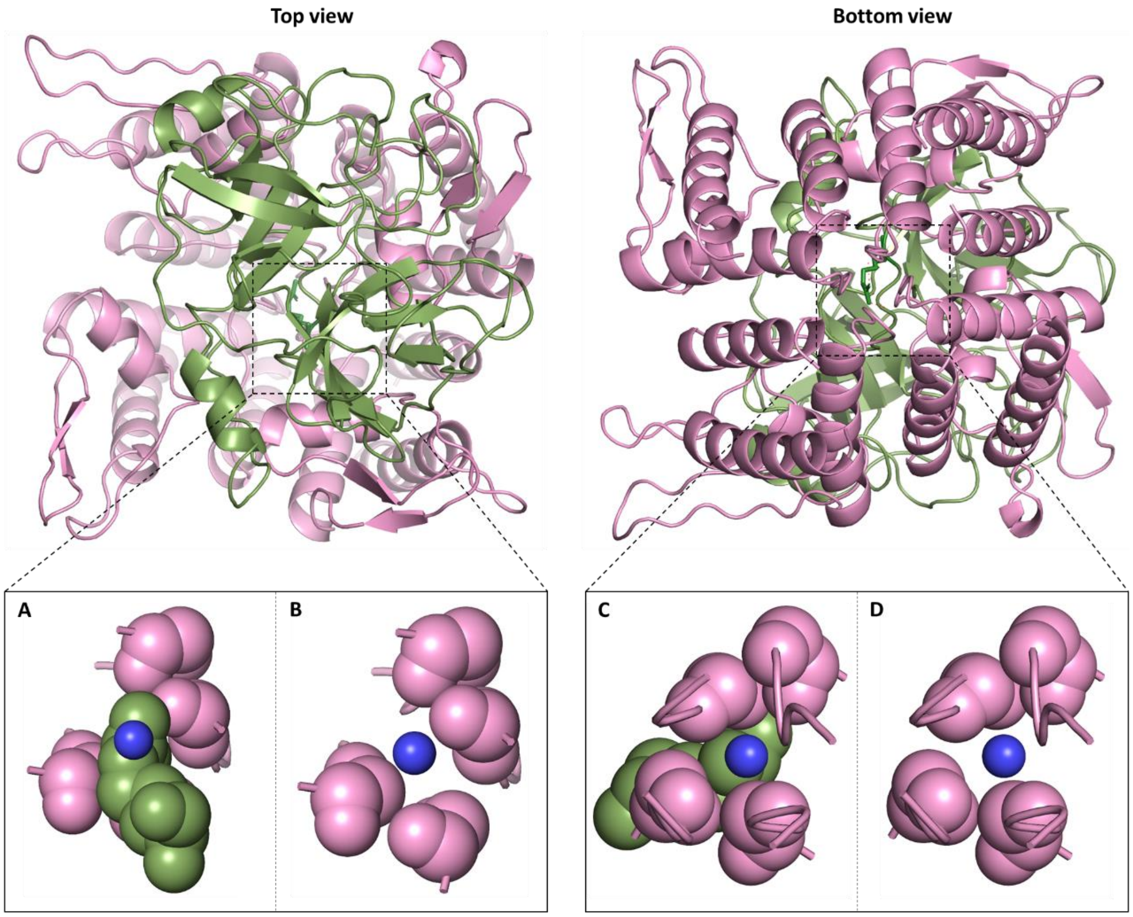

Figure S8. Top and bottom view of the selected docking solution for collinein-1 and hEAG1 channel. Zoomed panels of each structure show the blockage of the hEAG1 selectivity filter by Arg79 residue from collinein-1. Panels A and B represent, in a top view, the selectivity filter in the presence and in the absence of collinein-1, respectively. Panels C and D represent, in a bottom view, the selectivity filter in the presence and in the absence of collinein-1, respectively. Collinein-1 is represented as green cartoon and hEAG1 channel is represented in pink cartoon. Potassium ion is represented as a blue ball.

Fig S9.

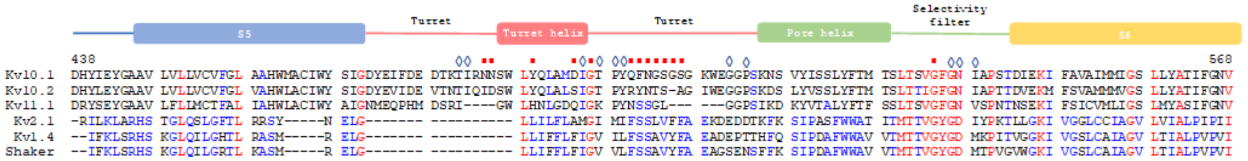

Figure S9. Multiple sequence alignment of hEAG1 channel region involved in the interaction interface with collinein-1. Helixes, loops and residues numbering are based on the hEAG1 structure. Amino acid residues that make hydrogen bonds with collinein-1 residues are indicated by the red box, while those involved in hydrophobic contacts with the toxin are indicated by the blue lozenge. Sequence accession numbers from NCBI GenBank database: human EAG1 (Kv10.1), NP_758872.1; human EAG2 (Kv10.2), NP_647479.2; human ERG1 (Kv11.1), XP_016867684.1; rat Kv2.1, NP_037318.1; rat Kv1.4, NP_037103.1; shaker, CAA29917.1.

Table S1. Comparison of participating residues of different serine proteases in the interaction with hEAG1, according to the proposed docking model. The residue numbers are related to their position at collinein-1 sequence, according to the alignment present in figure 3.

Residue	collinein-1	rCollinein-mut	gyroxin B1.3	BjSP	chymotrypsin
Ser25	•	•	•		
Phe26	•	•	•	•	
Arg46	•	•	•		
Lys47	•	•	•		
Lys71	•	•	•	•	
Glu72	•	•	•	•	
Lys73	•	•	•	•	
Tyr74	•	•	•		
Asn77	•	•	•		
Arg79	•	•	•		
Asn80	•	•	•		
Lys84	•	•	•		
Asn95	•	•	•		
Glu133	•	•	•		
Arg153	•	•	•	•	
Ala163	•	•	•	•	
Arg182	•	•	•		
Glu232	•	•	•		
Val234	•	•	•		
Cys236	•	•	•	•	
